# Antibacterial mechanism of the action of *Enteromorpha linza* L. essential oil against *Escherichia coli* and *Salmonella Typhimurium*

**DOI:** 10.1186/s40529-015-0093-7

**Published:** 2015-05-23

**Authors:** Jayanta Kumar Patra, Gitishree Das, Kwang-Hyun Baek

**Affiliations:** School of Biotechnology, Yeungnam University, Gyeongsan, 712-749 Gyeongbuk Republic of Korea

**Keywords:** Cell membrane, Enteromorpha linza, Escherichia coli, Essential oil, Salmonella Typhimurium, Seaweed

## Abstract

**Background:**

Identification of natural antibacterial agents from various sources that can act effectively against disease causing foodborne bacteria is one of the major concerns throughout the world. However, the natural antibacterial agents identified to date are primarily effective against Gram positive bacteria, but less effective against Gram negative bacteria. In the present study, *Enteromorpha linza* L. essential oil (EEO) was evaluated for antibacterial activity against *Escherichia coli* and *Salmonella Typhimurium* along with the mode of their antibacterial action.

**Results:**

The chemical composition of EEO revealed high amounts of acids (54.6 %) and alkenes (21.1 %). EEO was effective against both *E. coli* and *S. Typhimurium*. The minimum inhibitory concentration (MIC) and minimum bactericidal concentration (MBC) values of EEO for both pathogens were 12.5 mg/ml and 25.0 mg/mL, respectively. EEO at the MIC acted on the loss in viability of *E. coli* ATCC 43890, which was used as the model system for evaluation of the antibacterial mode of action of EEO against Gram negative bacteria. Significant increase in relative electrical conductivity and K^+^ concentration were recorded with respect to time, indicating the disruption of tested *E. coli* cells owing to the controlling effect of EEO. Alternation of the morphology of the cell surface, increase in the release of 260 nm absorbing materials and loss of high salt tolerance were observed.

**Conclusions:**

The results suggest that EEO induced a bactericidal effect via structural membrane damage caused by deposition of EEO in the cytosol or through enzymatic degradation of bacterial intracellular enzymes that resulted in cellular lysis. Accordingly, EEO can be used as a strong natural antibacterial agent against Gram negative foodborne pathogens such as *E. coli* and *S. Typhimurium*.

**Electronic supplementary material:**

The online version of this article (doi:10.1186/s40529-015-0093-7) contains supplementary material, which is available to authorized users.

## Background

Foodstuffs of animal and plant origin along with sea products are always subjected to spoilage and decaying phenomena that occur due to various microorganisms, mainly bacteria (Kim et al. [1995]; Faid [2011]). These phenomena often lead to heavy loss of food products and raw materials, ultimately resulting in health problems caused by the occurrence of food poisoning and infections (Kim et al. [1995]). Contamination of food by different types of microorganisms is a matter of great concern for this world with increasing human population day by day (Acharya et al. [2011]). Various foodborne microorganisms negatively affect food via two possible pathway, direct growth in food products and the release of microbial toxins while handling the food before processing. However, it is always difficult to find solutions to control food contamination.

The use of chemicals to protect meat, fruits, vegetables and drinks can be harmful to human health and the environment (Acharya et al. [2011]; Khwaldia [2011]). However, the application of chemical antimicrobials has been approved by different medical authorities worldwide to maintain food security. Owing to increased awareness, the use of synthetic chemicals has been rejected by consumers because of their adverse side effects that in many cases led to deadly diseases (Goni et al. [2009]). As a result, the demand for natural preservatives in food has increased, leading many food industries to search for natural antimicrobial agents for use in packaging and preservation. Indeed, the demand for natural antimicrobial agents is high as they are more effective and generally recognized as safe (GRAS) (Acharya et al. [2011]).

More than 150,000 algae and seaweed species exist in the ocean, many of which possess components with antibacterial, antifungal and antiviral properties (Salvador et al. [2007]; Souhaili et al. [2008]; Lategan et al. [2009]; Plaza et al. [2010]; Faid [2011]). Several chemical compounds from marine seaweeds such as agar, carrageenan, alginates and pigments like beta carotene have been used widely in food industries and pharmacology. Marine algae/seaweeds have become a part of the human diet in many countries, and many ingredients from these seaweeds have also being used in drinks, snacks, soups and beverages (Faid [2011]). Various natural antimicrobial agents that have the potential for food processing and preservation are effective against Gram positive foodborne bacteria, but they are very less effective and sometimes not effective against the Gram negative foodborne bacteria (Trombetta et al. [2005]; Bassole and Juliani [2012]; Sfeir et al. [2013]; Nazzaro et al. [2013]).

*Enteromorpha linza* (L.) is a common edible seaweed that is abundant in coastal areas of Asian and European countries (Say et al. [1986]) and consumed as food by people worldwide (Demirel et al. [2011]). Therefore, in this study, we evaluated the antibacterial potential of essential oil extracted from *E. linza* against two dominant Gram negative foodborne pathogenic bacteria, *Escherichia coli* and *Salmonella Typhimurium*, as well as its mode of antibacterial action.

## Methods

### Extraction of essential oil from *E. linza*

Fresh seaweed (*E. linza* L.) was purchased from a local market at Gyeongsan, Republic of Korea, washed thoroughly and put into a specially designed glass container. Five liters of water were then added to a glass container containing 500 g seaweed and subjected to hydro-distillation to extract essential oils using a micro-wave assisted extraction machine manufactured by KMD Engineering (Paju, Republic of Korea) for 4 h. The temperature of the chamber was maintained by a thermo controller (oven power capacity of 40 W and frequency of 15 gkH). Approximately 500 mL of the distillate was then collected from the collecting nozzle of the apparatus with a conical flask, mixed with an equal volume of dichloromethane in a separating funnel, vigorously shaken, and allowed to stand until the two layers separated. Next, the lower layer was taken and concentrated using a rotary evaporator (N-1110, Eyela, Tokyo Rikakikai Co., Ltd., Japan) at 40 °C. The extracted *E. linza* L. essential oil (EEO) was then kept in a tightly closed vial at 4 °C until further use.

### Chemical analysis of EEO

A detailed analysis of the chemical composition of EEO was conducted using a gas chromatography–mass spectrometry system (JMS 700 Mstation, Jeol Ltd., Japan) equipped with an Agilent 6890 N GC DB-5 MS fused silica capillary column (Agilent Technologies, Santa Clara, USA) according to the method described by Patra et al. ([2015]).

### Determination of antibacterial activity of EEO

The antibacterial potential of EEO was evaluated against four foodborne bacteria belonging to two species, *S. Typhimurium* ATCC 19586 and ATCC 43174 and *E. coli* ATCC 43889 and ATCC 43890. The pathogens were obtained from the American Type Culture Collection (ATCC, Manassas, VA, USA). Prior to use, EEO was diluted two times in 5 % dimethylsulphoxide (DMSO), then filter-sterilized using a 0.22 μm micro syringe filter (Chemco Scientific, Chungbuk, Republic of Korea). The standard disc diffusion method described by Diao et al. ([2013]) was employed to evaluate the antibacterial activity of EEO. EEO and rifampicin (Duchefa Biochemie, Haarlem, The Netherlands) at 25 mg/disc and at 20 μg/disc, respectively, were used as the test sample and the positive control, while 5 % DMSO was used as the negative control. The inhibition zone was measured in mm for each pathogen.

The minimum inhibitory concentration (MIC) and minimum bactericidal concentration (MBC) of EEO against the four pathogens was determined by the two-fold dilution method described by Kubo et al. ([2004]), with slight modification. The lowest concentration of EEO without any visible growth of test organism was taken as the MIC value, and the concentration not showing any growth of bacterial colony on nutrient agar (NA) was selected as the MBC. Both the MIC and MBC were expressed in mg/mL. All experiments were repeated three times.

### Effect of EEO on the viability of *E. coli*

The effects of EEO on the viability of Gram negative foodborne bacteria were investigated according to the procedure described by Joray et al. ([2011]). Only one foodborne bacterium, *E. coli* ATCC 43890, was selected as the model organism for all the subsequent assays. Overnight grown bacterial culture treated with EEO at the MIC served as the treatment, while those treated with 5 % DMSO served as the control. Both treatment and control samples were incubated at 37 °C for 8 h, during which time samples were collected every 2 h, appropriately diluted in phosphate buffer saline, spread on the surface of NA plates and incubated further at 37 °C for 24 h. After incubation, the number of bacterial colonies in terms of colony forming unit (cfu) was determined by multiplying it with the appropriate dilution factor. The results were expressed in terms of log_10_ (cfu/mL).

### Effect of EEO on surface morphology of *E. coli*

The effects EEO treatment on the morphology of *E. coli* ATCC 43890 were determined by scanning electron microscopy (SEM). Both a control treated with 5 % DMSO and sample treated with EEO were prepared according to the procedure described by Bajpai et al. ([2009]). The specimens were then sputter-coated with platinum in an ion coater for 120 s and observed under SEM (S-4100, Hitachi, Japan).

### Effect of EEO on cell membrane permeability of *E. coli*

The effect of EEO on the cell membrane permeability of *E. coli* ATCC 43890 were evaluated by the standard procedure (Kong et al. ([2008]). The loss in membrane permeability of the bacterial cell was measured using a conductivity meter (Con 6, LaMotte, Maryland, USA) and calculated in terms of its relative electrical conductivity according to the following formula:

$$\mathrm{Relative}\;\mathrm{conductivity}\;\left(\%\right)\;=\;\left[\left(L2\;–\;L1\right)/L0\right]\;X\;100$$

where, L_0_ was the electrical conductivity of the bacteria in 5 % glucose killed by being treated in boiling water for 5 min, L_1_ was the electrical conductivity of the bacteria in 5 % glucose with the EEO at the MIC and L_2_ was the electrical conductivity of the EEO-bacteria mixtures in 5 % glucose collected at 2 h intervals during 8 h of incubation.

### Effect of EEO on *E. coli* cell membrane integrity

The effect of EEO on the cell membrane integrity of *E. coli* ATCC 43890 were measured by the standard procedure described by Carson et al. ([2002]). Both the control treated with DMSO and the sample treated with EEO at the MIC were incubated at 37 °C. After 60 and 120 min, samples were centrifuged at 3500 rpm for 10 min and the absorbance of the supernatant at 260 nm was measured using a spectrophotometer (ASP 3700, ACTGene Inc., NJ, USA).

### Effect of EEO on the leakage of potassium ion from *E. coli*

The leakage of potassium ion (K^+^) in the suspension culture of *E. coli* ATCC 43890 treated with EEO at the MIC was measured according to the method described by Lee et al. ([2002]) using a Kalium/Potassium kit (Quantofix, Macherey-Nagel GmbH & Co., Germany). All measurements were carried at 2 h time intervals during 8 h of incubation and expressed as the amount of free K^+^ ion (mg/L) in the bacterial suspension at each time interval. Bacterial culture treated with DMSO was used as a control.

### Effect of EEO on salt tolerance capacity of *E. coli*

The effect of EEO on the salt tolerance capacity of *E. coli* ATCC 43890 were determined by growing the bacterial cultures on NA plates supplemented with different concentrations of NaCl (0, 2.5, 5.0 and 10.0 %) (Miksusanti et al. [2008]). Bacterial cultures amended with 5 % DMSO and EEO at the MIC were plated on NA plates and incubated at 37 °C for 24 h. After determining the CFU, the results were expressed in terms of Log_10_ (cfu/mL).

### Statistical analysis

The results of all experiments were expressed as the mean ± standard deviation (SD). Statistical analyses consisted of one way ANOVA and Duncan’s multiple range tests, with *P <* 0.05 considered to indicate significance. Statistical Analysis Software (SAS) version 9.4 (SAS Inc., Cary, USA) was used for all analyses.

## Results and discussion

For years, pathogenic bacteria have been known to be the primary cause of foodborne illness and food poisoning throughout the world (Kim et al. [1995]). The use of natural antibacterial agents in food processing and food preservation has played a vital role in controlling the adverse effect of bacteria in food. However, the natural antibacterial agents currently used as preservatives are primarily effective against Gram positive foodborne pathogens, whereas they are not much effective against the Gram negative foodborne pathogens (Trombetta et al. [2005]; Bassole and Juliani [2012]; Sfeir et al. [2013]; Nazzaro et al. [2013]). The reason for the failure of most natural antibacterial agents against Gram negative pathogens is that they are unable to penetrate the strong cell wall of a Gram negative bacterium and thus do not affect it (Trombetta et al. [2005]).

There have been many reports of the bioactive potential of marine algae and seaweeds, and many novel compounds have been isolated from them (Kamat et al. [1992]; Crasta et al. [1997]; Bhosale et al. [2002]; Salvador et al. [2007]; Lategan et al. [2009]; Patra et al. [2009]; Plaza et al. [2010]). However, there have been limited reports on the extraction and evaluation of bioactive potential of essential oils from marine algae and seaweeds on antibacterial capacity. In this study, we evaluated the marine resource *E. linze* as a source of essential oil for use as a strong antibacterial agent that can efficiently control against the Gram negative bacteria *E. coli* and *S. Typhimurium*.

The composition of essential oil obtained from hydro-distillation of *E. linze* was analyzed by GC-MS analysis and individual compounds present in the EEO were described in detail in our earlier publication (Patra et al. [2015]). All of the identified compounds representing 94 % of the oil were categorized into six different groups (Table 1), 30 % of which had antimicrobial potential. Acids (54.6 %) and alkenes (21.1 %) were found in higher quantities, followed by alcohols (4.5 %), aldehydes (3.7 %) and ketones (2.8 %) (Table 1). The compounds with antimicrobial potential as previously reported include n-hexanal, 2-chloro-5-methyl-4-(2-thienyl) pyrimidine, tetradecanoic acid, pentadecanoic acid, (z,z)-6,9-cis-3,4-epoxy-nonadecadiene, 13-octadecenal and azetidine (Gutierrez et al. [2002]; Agoramoorthy et al. [2007]; Deep et al. [2012]; Jemaa [2014]; Sharma et al. [2014]).

**Table 1:** Percentage composition of functional groups present in *Enteromorpha linza* essential oil

Functional group	Active compounds	Percentage composition
Acid	Cyclopropanecarboxylic acid	54.6
	Tetradecanoic acid	
	Pentadecanoic acid	
	Pentadecanoic acid	
	Hexadecanoic acid	
Alkene	1-heptadecene	21.1
	(z,z)-6,9-cis-3,4-epoxy-nonadecadiene	
Alcohol	2-Hexanol	4.5
	Tridecanol	
Aldehyde	n-Hexanal	3.7
	2,4 heptadienal	
	2 Octenal	
	n-nonanal	
	13-Octadecenal	
Ketone	2-heptanone	2.8
	Alpha-Ionone	
	Trans-beta-Ionone	
	2(4H)-Benzofuranone	
Others	Dimethyl sulfone	6.9
	2-chloro-5-methyl-4-(2-thienyl) pyrimidine	
	2-pentadecen-4-yne	
	3-heptadecen-5-yne	
	Azetidine	

The antibacterial potential of EEO against four foodborne bacterial pathogens is presented in Table 2. The result showed that EEO was most effective against *E. coli* ATCC 43889, with a 13.33 mm inhibition zone, as well as against all tested *S. Typhimurium* strains, with a 10.66 mm inhibition zone. Rifampicin, a standard antibiotic showed significantly high controlling activity against all four strains of the tested bacteria, whereas DMSO, a negative control) exerted no inhibitory activity. The MIC values of the EEO ranged from 12.5 to 25 mg/mL, whereas it had an MBC of 25 mg/mL against all four test pathogens (Table 3).

**Table 2:** Efficacy of *Enteromorpha linza* essential oil (EEO) against four strains of the two Gram negative foodborne pathogens, *E. coli* and *S. Typhimurium*

Foodborne bacterial pathogen	Zone of inhibition (mm)	
	EEO^#^	Rifampicin^##^
*E. coli* ATCC 43889	13.33 ± 0.58^a^	32.33 ± 1.52^a^
*E. coli* ATCC 43890	10.00 ± 0.00^b^	32.66 ± 0.57^a^
*S. Typhimurium* ATCC 19586	10.66 ± 1.15^ab^	21.50 ± 4.94^b^
*S. Typhimurium* ATCC 43174	10.66 ± 0.57^ab^	31.66 ± 2.08^a^

Values are expressed as the mean ± standard deviation. Values with different superscript letters in the same column are significantly different at *P <* 0.05

^#^EEO at 25 mg/disc; ^##^Rifampicin as a standard antibiotic at 20 μg/disc

**Table 3:** MIC and MBC values of *Enteromorpha linza* essential oil against four strains of two Gram negative foodborne pathogens

Foodborne bacterial pathogen	MIC (mg/mL)	MBC (mg/mL)
*E. coli* ATCC 43889	12.5	25.0
*E. coli* ATCC 43890	12.5	25.0
*S. Typhimurium* ATCC 19586	25.0	25.0
*S. Typhimurium* ATCC 43174	12.5	25.0

Only a few studies have investigated the antibacterial effects of algal essential oil on pathogenic bacteria (Koz et al. [2009]; Demirel et al. [2011]; Gressler et al. [2011]). However, the results of the present study clearly demonstrated that EEO exerted antibacterial effects on Gram negative bacteria. The outer membrane of Gram negative bacteria is always impermeable to various antibacterial agents owing to their strong lipolysaccharide molecules (Bezic et al. [2003]; Sharma et al. [2013]). In this study, however, the positive results of antibacterial activity of EEO against the two Gram negative bacteria suggested an hypothesis that, although essential oil is hydrophobic in nature, it might have slowly entered into the bacterial cell through the general bacterial porins owing to its small size, after which it might have affected the bacterial enzymes, causing cellular lysis and death (Nikaido [1996]; Kotzekidou et al. [2008]). The antibacterial activity of the EEO against the tested pathogenic bacteria might be due to the presence of various active compounds such as n-hexanal, 2-chloro-5-methyl-4-(2-thienyl) pyrimidine, tetradecanoic acid, pentadecanoic acid, (z,z)-6,9-cis-3,4-epoxy-nonadecadiene, 13-octadecenal and azetidine in the EEO that might have individually of synergistically altogether inhibited the growth of the pathogenic bacteria.

**Fig. 1:**
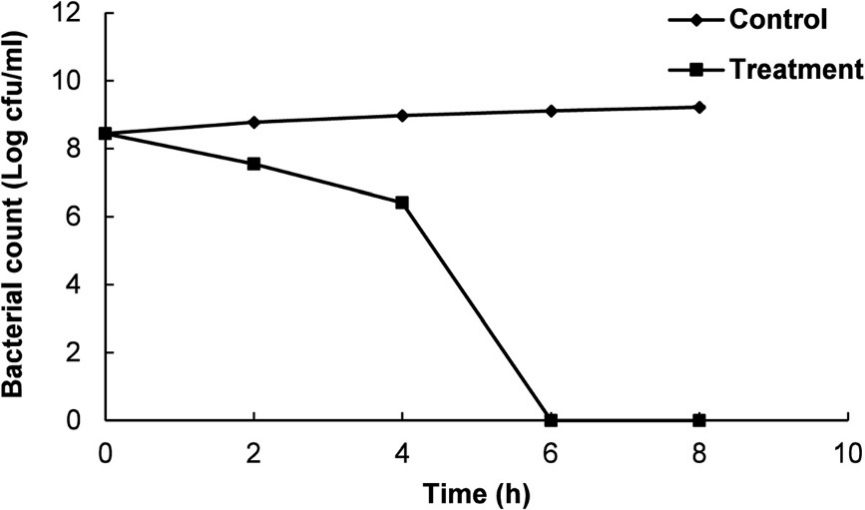
Effect of *Enteromorpha linza* essential oil on the cell viability of *E. coli* ATCC 43890. The control and treatment were *E. coli* ATCC 43890 treated with 5 % DMSO and *Enteromorpha linza* essential oil at the MIC, respectively. Values are expressed as the mean ± SD

Among the two investigated pathogens, EEO was more effective against *E. coli* (Tables 2 and 3); hence, *E. coli* ATCC 43890 was selected for further investigation of its antibacterial mode of action. The antibacterial mode of action of EEO against *E. coli* ATCC 43890 was evaluated by several assays, including cell viability, observation by SEM, effect on cell membrane leakage and loss of salt tolerance. The effects of EEO on the viability of *E. coli* ATCC 43890 are shown in Fig. 1. EEO at the MIC gradually decreased the viability of the bacterial cells by 4 h of incubation; however, it drastically decreased the number of CFU between 4 and 6 h, and completely inhibited cell growth after 6 h of incubation. In contrast, there was a continuous increase in the number of CFUs on the control plates.

**Fig. 2:**
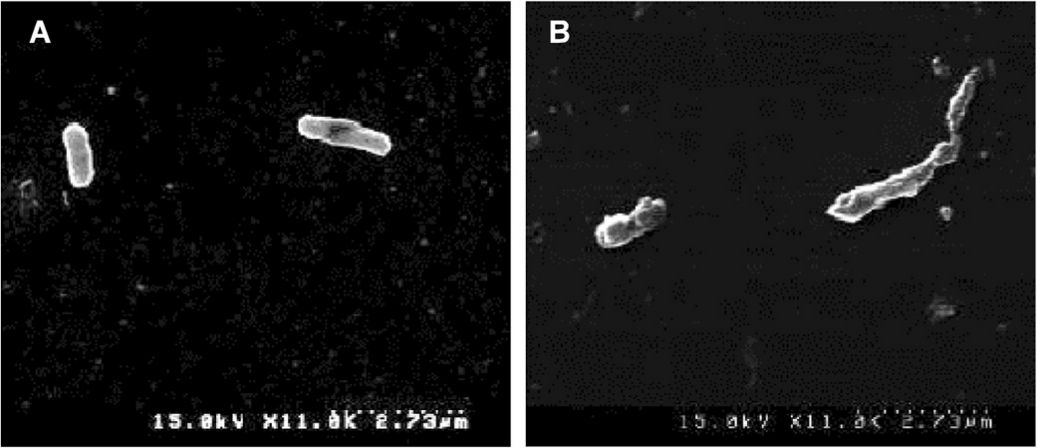
Photograph of the scanning electron microscopy image of *E. coli* ATCC 43890. **a**: *E. coli* treated with 5 % DMSO; **b** *E. coli* treated with *Enteromorpha linza* essential oil at the MIC

Disruption of the morphology of *E. coli* ATCC 43890 by EEO was also observed by SEM analysis (Fig. 2). When compared with the uniform and smooth surface found in the control *E. coli* treated with 5 % DMSO, a ruptured and rough surface with an elongated morphology was observed in bacterial cells treated with EEO at the MIC. As shown in the mode of action for other essential oils, EEO might have induced a bactericidal effect through membrane damage and enzymatic degradation that resulted in cellular lysis (Ghannoum [1988]; Bajpai et al. [2009]).

**Fig. 3:**
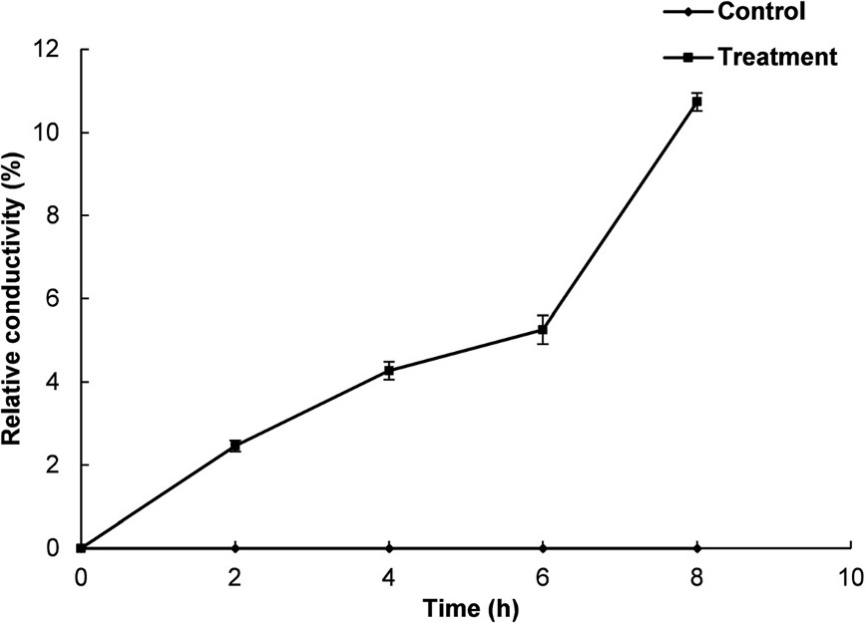
Effect of *Enteromorpha linza* essential oil at the MIC on the membrane permeability of *E. coli* ATCC 43890. The control and treatment were *E. coli* ATCC 43890 treated with 5 % DMSO and *Enteromorpha linza* essential oil at the MIC, respectively. Values are expressed as the mean ± SD

**Fig. 4:**
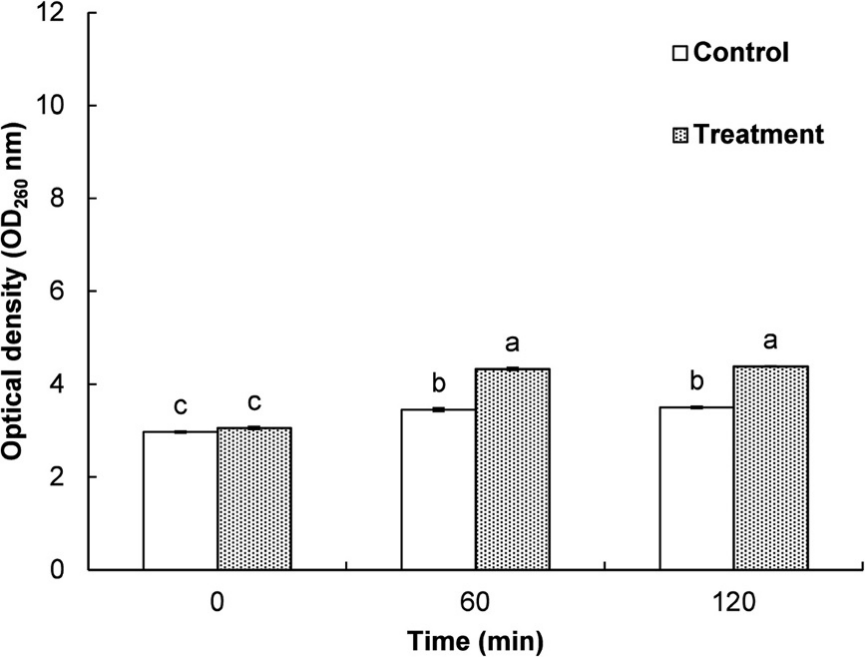
Effect of *Enteromorpha linza* essential oil at the MIC on the release rate of 260 nm absorbing material from *E. coli* ATCC 43890. The control and treatment were *E. coli* ATCC 43890 treated with 5 % DMSO and *Enteromorpha linza* essential oil at the MIC, respectively. Data are expressed as the mean ± SD. Values with different superscript letters are significantly different at *P <* 0.05

**Fig. 5:**
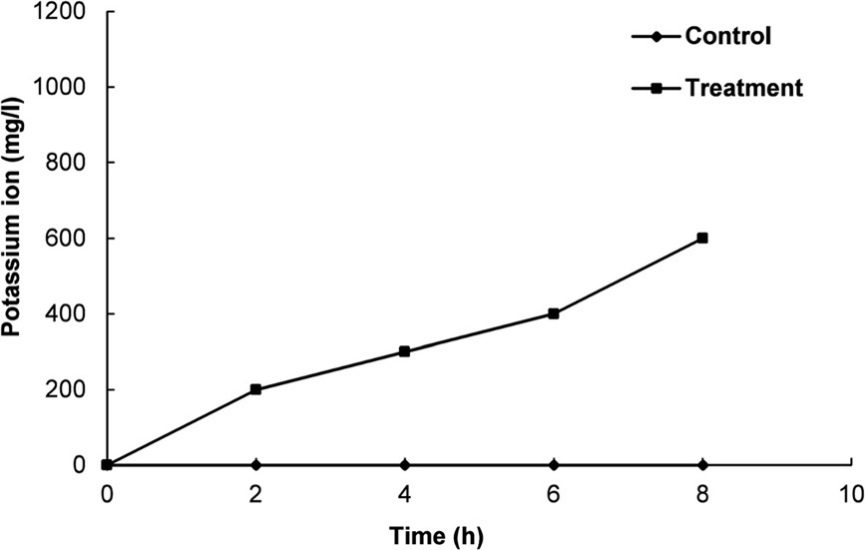
Effect of *Enteromorpha linza* essential oil at the MIC on the leakage of potassium ions from *E. coli* ATCC 43890. The control and treatment were *E. coli* ATCC 43890 treated with 5 % DMSO and *Enteromorpha linza* essential oil at the MIC, respectively

The effect of EEO and DMSO on the membrane permeability of *E. coli* ATCC 43890 are presented based on the relative conductivity, optical density and free K^+^ contents (Figs. 3, 4 and 5). The control sample treated with DMSO showed no increase in the relative electrical conductivity with respect to time, whereas *E. coli* ATCC 43890 treated with EEO showed increased relative electrical conductivity with respect to time (Fig. 3). Especially, after 6 h of incubation, there was a sharp increase in the relative electrical conductivity (Fig. 3). The effect of EEO at the MIC on the release of 260 nm absorbing materials from *E. coli* ATCC 43890 are presented in Fig. 4. *E. coli* ATCC 43890 treated with EEO at the MIC displayed a continuous increase in the optical density; however, the control treated with 5 % DMSO showed no remarkable change in optical density (Fig. 4). *E. coli* ATCC 43890 treated with EEO at the MIC displayed a continuous increase in the leakage of K^+^ ions from bacteria with respect to incubation time. An especially sharp increase in the leakage of K^+^ was observed after 6 h of incubation (Fig. 5), corresponding to the complete loss of cell viability and a sharp increase in relative electrical conductivity in *E. coli* ATCC 43890 treated with EEO at the MIC after 6 h of incubation (Figs. 1 and 3).

The increase in the relative electrical conductivity, leakage of 260 nm absorbing materials and leakage of K^+^ ions to the incubating culture indicated that EEO after being escaped into the bacterial cells through the general porins might have been accumulated in the cytosol, resulting in loss of plasma membrane integrity. This caused the cells to become permeable to protons and ions from outside, which eventually caused cellular lysis and cell death (Bajpai et al. [2013]). The loss of 260 nm absorbing materials along with the leakage of K^+^ ions to the outside of the bacteria might have been due to the structural damage caused by the action of EEO (Cox et al. [2001]). In addition, the loss of membrane permeability, release of 260 nm absorbing material, and leakage of K^+^ ions to the outside can be sensitive indicators of the disruption of membrane integrity (Cox et al. [2001]). Any type of minor changes to the structural integrity of the cell membrane could adversely affect the normal metabolic functioning of the bacterial cell and result in complete lysis (Hugo and Snow [1981]; Davidson and Branen [2005]).

**Fig. 6:**
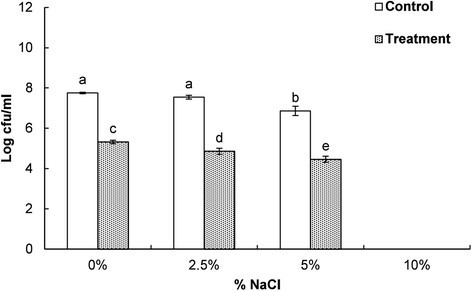
Effect of *Enteromorpha linza* essential oil at the MIC on the loss of salt tolerance capacity of *E. coli* ATCC 43890. The control and treatment were *E. coli* ATCC 43890 treated with 5 % DMSO and with *Enteromorpha linza* essential oil at the MIC, respectively. Data are expressed as the mean ± SD. Values with different superscript letters are significantly different at *P <* 0.05

Loss of the ability to tolerate high salt concentrations was observed in *E. coli* ATCC 43890 treated with EEO at the MIC (Fig. 6). When the *E. coli* strain pretreated with EEO at the MIC was incubated on NA supplemented with different concentrations of NaCl (0, 2.5 and 5 %), its concentration decreased significantly relative to bacterium pretreated with 5 % DMSO (Fig. 6). The underlying mechanism for the loss of salt-tolerance capacity might have been related to the loss of membrane permeability and degradation of important enzymes inside the bacterial cells. Additionally, EEO might have caused reduced osmoregulatory ability in high salt concentrations, resulting in their loss of salt tolerance (Miksusanti et al. [2008]).

## Conclusions

EEO from the edible marine seaweed *E. linza* was found to have a potential inhibitory effect on two Gram negative foodborne bacteria. This effect of EEO was associated with its ability to disrupt the bacterial membrane and probably the bacterial enzymes by accumulation inside the cytosol, causing loss of membrane integrity and leakage of 260 nm absorbing material along with K^+^ ions. Since EEO is extracted from the edible seaweed widely available around the coastal belt of many countries worldwide, it can be an inexpensive but valuable source of natural mixtures of antibacterial compounds that exhibit the potential for use in food systems. EEO could prevent the growth of foodborne bacteria, particularly Gram negative bacteria, and extend the shelf life of the processed food.

## Authors’ information

Jayanta Kumar Patra and Gitishree Das were the combined first authors to the manuscript.

## Authors’ original submitted files for images

Below are the links to the authors’ original submitted files for images. Authors’ original file for figure 1 Authors’ original file for figure 2 Authors’ original file for figure 3 Authors’ original file for figure 4 Authors’ original file for figure 5 Authors’ original file for figure 6
