# Supplementary figures and images for: Antibacterial mechanism of the action of Enteromorpha linza L. essential oil against Escherichia coli and Salmonella Typhimurium
Source: Bot Stud. 2015 May 23;56:13. doi: 10.1186/s40529-015-0093-7 (PMC5432928; doi:10.1186/s40529-015-0093-7)

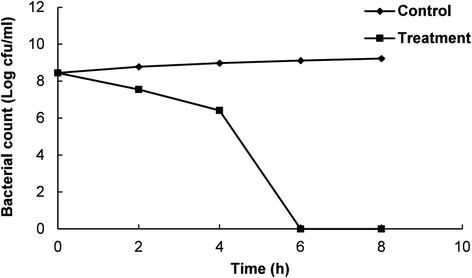

Supplement: Supplementary file 1 — Authors’ original file for figure 1 [file 40529_2015_93_MOESM1_ESM.gif]

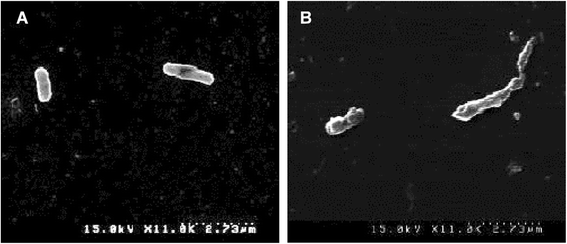

Supplement: Supplementary file 2 — Authors’ original file for figure 2 [file 40529_2015_93_MOESM2_ESM.gif]

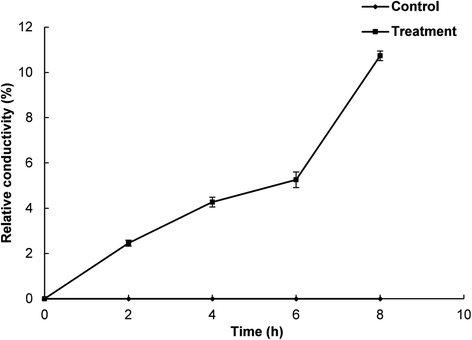

Supplement: Supplementary file 3 — Authors’ original file for figure 3 [file 40529_2015_93_MOESM3_ESM.gif]

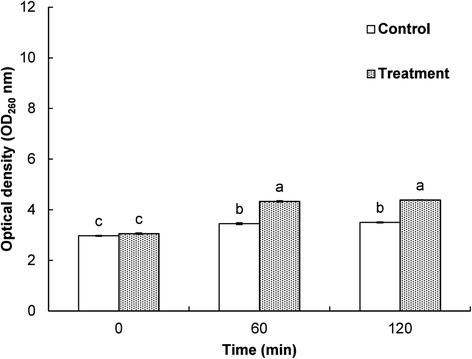

Supplement: Supplementary file 4 — Authors’ original file for figure 4 [file 40529_2015_93_MOESM4_ESM.gif]

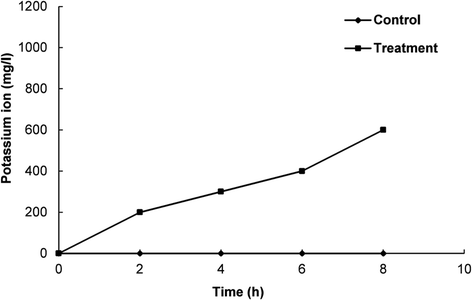

Supplement: Supplementary file 5 — Authors’ original file for figure 5 [file 40529_2015_93_MOESM5_ESM.gif]

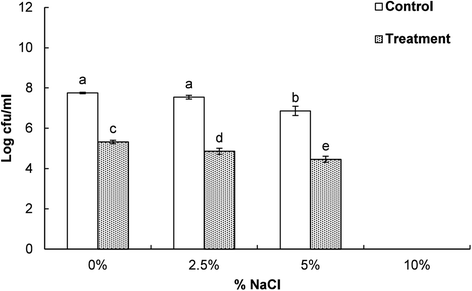

Supplement: Supplementary file 6 — Authors’ original file for figure 6 [file 40529_2015_93_MOESM6_ESM.gif]
